# SENSORY LOSS AND THE HEALTH CARE SYSTEM

**DOI:** 10.1093/geroni/igz038.166

**Published:** 2019-11-08

**Authors:** Nicholas Reed, Charlotte Yeh

**Affiliations:** 1 Johns Hopkins University, Baltimore, Maryland, United States; 2 AARP Services, Inc., Washington, District of Columbia, United States

## Abstract

Communication is fundamental to patient-centered care. However, sensory impairment may limit communication among older adults. Specifically, hearing impairment strains communication via degraded auditory encoding while vision impairment distresses ability to read and interpret visual cues. The presence of dual sensory impairment, defined as concurrent hearing and vision impairment, may exacerbate these effects. The potential consequences of age-related sensory loss on health care interactions and outcomes are beginning to surface in epidemiologic studies demonstrating poorer patient-provider communication, higher incurred health care costs, increased risk of 30-day readmission, and longer length of stay when compared to individuals without sensory loss. Importantly, these associations may be amenable to intervention via sensory aids; however, uptake to sensory care is low. Notably, less than 20% of persons with hearing impairment have hearing aids and over 55% of Medicare Beneficiaries with reported vision problems have not had an eye examination in the prior year. Affordability and access may contribute to lack of sensory care uptake as Medicare explicitly excludes coverage of vision and hearing services. In this symposium, we will review current and new evidence for whether sensory loss affects health care outcomes, including satisfaction with care and medical costs, and present data on how persons with sensory loss interact with the health care system based on the need and reasons for accompaniment to care visits. Further, we will discuss and provide evidence for how sensory care may mitigate these associations. Lastly, we will place these results within the context of quality care and policy initiatives.

